# Development of scales to measure Lebanese university students’ perceived knowledge about and attitudes about cannabis use: initial psychometric properties

**DOI:** 10.1186/s42238-022-00144-x

**Published:** 2022-07-02

**Authors:** Anthony Mina, Clara Rahme, Souheil Hallit, Michel Soufia

**Affiliations:** 1 School of Medicine and Medical Sciences, Holy Spirit University of Kaslik, P.O. Box 446, Jounieh, Lebanon; 2 Psychology Department, College of Humanities, Effat University, Jeddah, 21478 Saudi Arabia; 3 Research Department, Psychiatric Hospital of the Cross, Jal Eddib, Lebanon

**Keywords:** Cannabis, Perceived knowledge, Attitude, University students, Lebanon

## Abstract

**Background:**

For a long period, cannabis in Lebanon was categorized as an illegal psychoactive substance criminalized by law, despite being the 3rd largest producer of cannabis resin after Morocco and Afghanistan. The current available literature on the topic in Lebanon is scarce, as perceived knowledge towards cannabis use in the general population is not well established, and public health policies are absent. In the context of future legalization of cannabis for both medical and recreational purposes, what is the public’s perception of cannabis perceived knowledge about and attitude against cannabis use? The objectives of this study were to create two scales to assess perceived knowledge towards cannabis use and attitude against cannabis use and check their psychometric properties in a sample of Lebanese university students.

**Methods:**

The study was designed as a cross-sectional study involving 415 Lebanese university students aged between 18 and 30 years. Students were only recruited from one university and were sent an email with a brief description of the study and a link to the questionnaire. The questionnaire items covered perceived knowledge and attitude about cannabis use, recovered and adapted from available literature.

**Results:**

Perceived knowledge items converged over a solution of four factors that had an Eigenvalue over 1, explaining a total of 64% of the variance (factor 1 = perceived knowledge about cannabis quality of life improvement; factor 2 = perceived knowledge about cannabis psychiatric use; factor 3 = perceived knowledge about cannabis risks; factor 4 = perceived knowledge about cannabis neurological use). Attitude items converged over a solution of two factors that had an Eigenvalue over 1, explaining a total of 58.2% of the variance (factor 1 = negative attitude about cannabis use; factor 2 = positive attitude about cannabis use). Good Cronbach’s alpha values were found for the perceived knowledge and attitude scales (*α* = 0.78 and *α* = 0.73 respectively). Greater perceived knowledge was found in male participants and in those who have tried cannabis for recreational purposes. Greater perceived knowledge was also associated with stronger attitude about cannabis use and older age. Furthermore, greater mean attitude about cannabis use scores were significantly found in male participants and in those who have tried cannabis for recreational or medicinal purposes.

**Conclusion:**

Primary results showed acceptable psychometric properties for the perceived knowledge towards and attitudes about cannabis use scale. Further studies are needed in order to validate them among the general population and assess more psychometric properties of these scales.

## Background

The United Nations Office on Drugs and Crime (UNODC) ranked Lebanon as the world’s third largest producer and supplier of cannabis resin in 2019 after Morocco and Afghanistan (World Drug Report 2018). Cannabis history and social background in Arabic-speaking countries date back to the ninth century when it was used to treat ear diseases (Fakhry et al. 2021). Cannabis was later employed by Arabic physicians for a variety of purposes such as anti-parasitic characteristics, dermatologic problems, and rheumatological/ophthalmological conditions (Lozano 2001). Cannabis was banned in Lebanon; nevertheless, it was widely grown, covering an estimated 400,000 hectares and centered mostly in the Beqaa valley, due to its unique geographic location and Mediterranean weather. Furthermore, its proximity to the borders, as well as the ease of access to trafficking routes, resulted in high-quality cultivation (Fakhry et al. 2021).

A draft law to legalize cannabis was released for parliament voting on October 17, 2019. Aside from the financial benefits of taxing and regulation, the goal of this law was to lessen the strain on the justice system by controlling product quality and safety and raising awareness about the negative health effects of cannabis usage (El-Khoury et al. 2020). Prior to the voting assembly, the Lebanese Psychiatric Society, which is accredited by the Lebanese Order of Physicians, issued a statement urging all involved parties to reconsider the proposed legalization of cannabis cultivation for medical purposes, as it does not guarantee that the local prevalence of cannabis use disorders will not rise. Similarly, the Lebanese National Ethics Committee advised that medical cannabis should be limited to situations where benefit has previously been shown, such as chemotherapy-induced nausea, HIV-related appetite loss, and multiple sclerosis spasticity (El-Khoury et al. 2020).

**Fig. 1:**
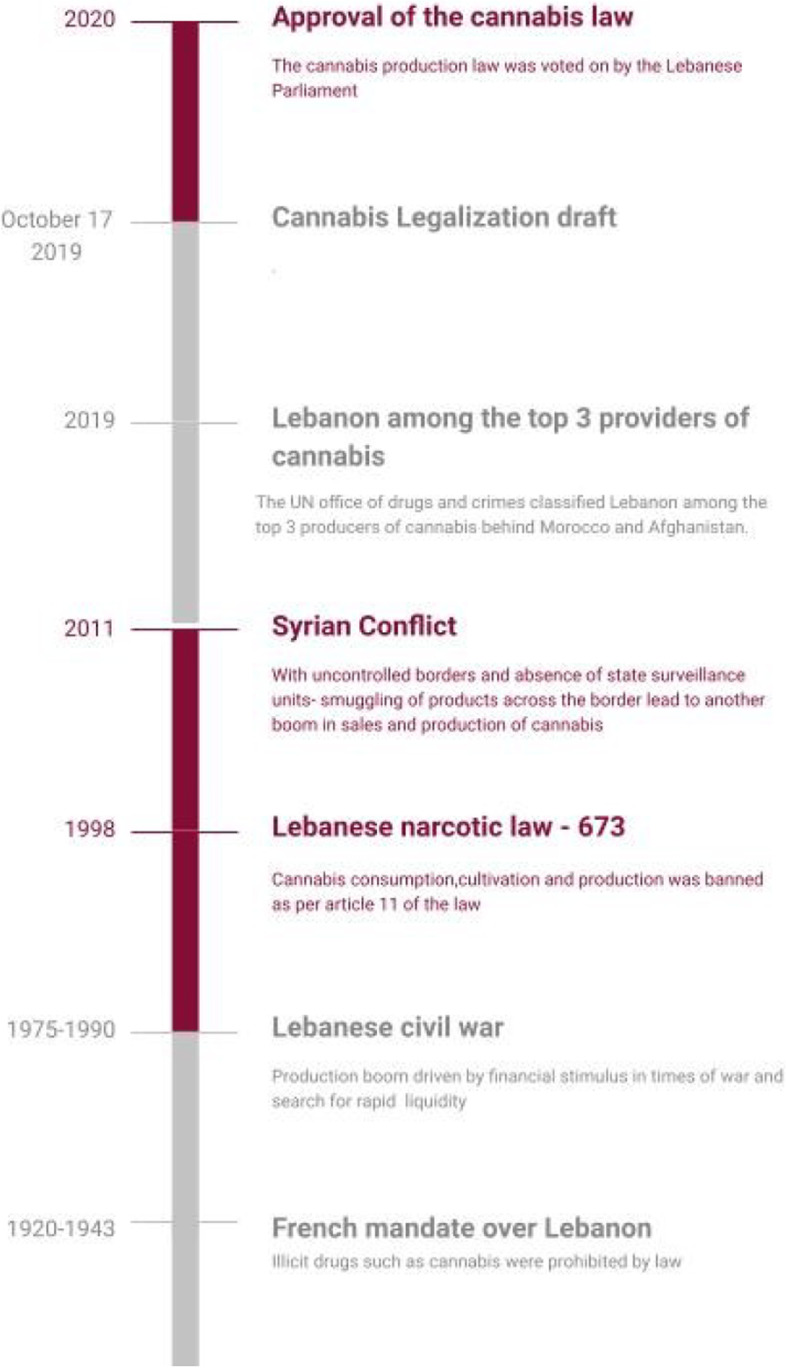
Cannabis historical timeline in Lebanon

The law was finally passed by the Lebanese Parliament on April 21, 2020 (Cannabis law and legislation in Lebanon [https:, , cms.law, en, int, expert-guides, cms-expert-guide-to-a-legal-roadmap-to-cannabis, lebanon]. n.d.). However, little was done to address local cannabis consumption because the law only permitted exportation under official authorization, leaving local medicinal consumption illegal and penalized. Indeed, under Lebanese law, items containing more than 1% tetrahydrocannabinol (THC) will remain illegal. More local information on the cannabis timeline can be found here in Fig. 1 (Cannabis law and legislation in Lebanon [https:, , cms.law, en, int, expert-guides, cms-expert-guide-to-a-legal-roadmap-to-cannabis, lebanon]. n.d.).

Under these circumstances, perceived knowledge toward and attitude about cannabis use became a great concern. Various studies underlined the moderating effect of perceived knowledge and risk perceptions on substance use, whereas a substance being considered as risky/dangerous is less likely to be used/abused (Salloum et al. 2018). This risk perception could be influenced by accumulated personal perceived knowledge and attitude on cannabis, hence affecting the decision of cannabis use in the general population (Park et al. 2020). Indeed, cannabis education has been shown to have a significant impact on using cannabis in the future (Okaneku et al. 2015), as seen in the National Survey on Drug use and health, whereas the states with its highest cannabis risk education presented a lower percentage of cannabis use among individuals (Hughes et al. 2016).

According to the Theory of Reasoned Action, attitude will be used to assess an individual’s beliefs and cognitive rational behaviors on cannabis use. The desired goal is to measure the overall attitude (positive vs. negative), which will drive an individual’s behavioral intentions and, in this case, the individual’s own behavior in relation to cannabis (Prochaska et al. 2002). In case of perceived knowledge, its intended use could be a deciding element in an individual’s personal decision to use cannabis, thereby contributing to the rational action pathway. Perceived knowledge will examine the reasoning elements and traits that form the foundations of each person’s decision-making process (Prochaska et al. 2002).

In Lebanon, a recent study in community pharmacists found that only 51% were found to have good perceived knowledge of cannabis uses and adverse effects as well as 44.7% were with legalization of cannabis in the country (Jaffal et al. 2020). In addition, among medical students, only 32.6% knew the adverse events of cannabis use (Assaf et al. 2017).

Studies assessing cannabis in any setting in the Arab and Lebanese region are relatively scarce, with few taking cannabis as the core substance in their study. Previous findings indicated that cannabis was the most used substance among high school and university students (Karam et al. 2010). Other ones indicated a 12.5% consumption rate of cannabis among university students, with 1 in 5 students likely to report a lifetime use of cannabis (Salameh et al. 2015; Ghandour and Donna S: El Sayed, and Silvia S. Martins. 2012).

To the best of our knowledge, previous studies have used various questionnaires to assess those variables but there are no validated scales for assessing perceived knowledge about and attitude about cannabis use. However, assessing such elements is critical since greater perceived knowledge and negative attitude entail better protection against cannabis abuse/misuse (Zvonarev et al. 2019). While this increase could be due to multiple reasons such as abolished legal penalty, decrease in prices and increased availability (various forms of cannabis products), and increased presence in daily life (social media, advertisement etc.), the health impact of legalization should be monitored extensively (Zvonarev et al. 2019). The current available literature on the topic in Lebanon is poor, as perceived knowledge about cannabis use in the general population is not well established, and public health policies are practically absent. In face of future legalization of cannabis use for both medical and recreational use, where does the public stand on the matter in terms of perceived knowledge and attitude? Going down the legalization road should be prepared by studies of the population mostly at risk: the young adult population, as a decriminalized and easier access will possibly lead to a surge in users and an increased exposure to cannabis products. The objectives of this study were to create and validate two scales to assess perceived knowledge towards cannabis use and attitude about cannabis use and check their psychometric properties in a sample of Lebanese university students.

## Methods

### Study design and participants

This is a cross-sectional study involving a sample of Lebanese university students aged between 18 and 30 years. The data collection was carried out during the month of January 2021. Excluded from this study were adults outside this age interval and those not currently enrolled in any major. The choice of sample is based on cannabis consumption being prevalent mostly in this age interval (Academies and of Sciences E, Medicine: The health effects of cannabis and cannabinoids: the current state of evidence and recommendations for research 2017; Keethakumar et al. 2021) and on cannabis being the second most used substance in Lebanese youth after alcohol (Salameh et al. 2015).

### Procedure

The coronavirus pandemic challenged the proper execution of the study due to nationwide lockdown, curfews, required social distancing, universities shutting down, and moving their presence to social media and online platforms. In response to this new situation, a Google Forms link was created. Prior to proceeding with the data collection, a pilot study consisting of 20 participants was conducted to assess the duration and technical feasibility and rephrase misunderstood questions. The data related to the pilot study was not included in the final database. Students were exclusively part of one university, recruited via an email containing a brief description of the study with the link of the questionnaire. The university in question requires its students to have an acceptable university level of English (reading/writing/speaking) prior to enrollment.

### Minimal sample size calculation

According to Comrey and Lee, a minimum of 10 observations is needed for each item of a scale intended to be validated. Since there were 29 items included in the perceived knowledge scale, a minimum number of 290 participants was deemed necessary. At the end of the data collection, 415 individuals responded to the questionnaire.

### Questionnaire

The questionnaire was available in English and was divided into 4 sections:

The first section contained a brief introduction of the study design, objectives, statement of privacy, and consent request from each participant before proceeding to the questions in the next section. The student had the choice to accept or refuse participation in this study.

The second section contained socio-demographic questions (such as age, gender, marital status), self-reported evaluation of physical activity index, and mental health (where the participant is simply asked to self-evaluate his/her current mental health status with responses varying from poor to excellent).

The third section included a yes/no type of questions related to cannabis use for recreational purposes: “Have you ever tried cannabis for recreational uses? and “Have you ever tried cannabis during the ongoing COVID-19 pandemic?”.

Moreover, participants were asked 16 different statements assessing their attitude about recreational use of cannabis in daily life, with responses varying from 1 = strongly agree to 5 = strongly disagree. These questions were adapted from other studies (Swift et al. 2005; Philpot et al. 2019) and relate mostly to driving under influence of cannabis, the frequency of cannabis use in the participant’s surrounding environment, access, and legal status of cannabis in the participant opinion. The total score varied between 16 and 80. Greater scores indicated stronger attitude about cannabis use.

The fourth section evaluated the participant’s perceived knowledge regarding cannabis utilization for medical purposes. This section first assessed the participant’s previous experience regarding medicinal cannabis using a yes/no type of question. Furthermore, a total of 29 questions evaluated cannabis effect on quality of life, utilizations in neurological and psychological scenarios, and assessment of the risks associated with its consumption. All questions were graded on a 5-point system with statements varying from strongly disagree to strongly agree. Scores varied between 29 and 145, with greater scores indicating greater perceived knowledge of medical cannabis applications and risks. The statements and questions forming this questionnaire were assembled from various studies assessing cannabis consumption in different populations (Swift et al. 2005; Philpot et al. 2019) and were tailored to the current population. Throughout the questionnaire, the authors referred to a reverse scoring system for some items in both scales.

### Statistical analysis

No missing data was found since all questions were required in the Google form. The FACTOR software was used to conduct the exploratory factor analysis of the perceived knowledge and attitude scales, using the Pearson correlation matrix and using the parallel analysis as a procedure for determining the number of factors/components. The promax rotation was used to extract items since the latter were highly correlated. The Kaiser–Meyer–Olkin (KMO) and the Bartlett’s test of sphericity *p*-value were calculated to ensure model’s adequacy. Factors with an Eigenvalue over 1 were retained; according to Kaiser (1960), there are as many reliable factors as there are eigenvalues greater than one. The reasoning is that an eigenvalue less than one implies that the scores on the component would have negative reliability (Cliff 1988; Kaiser 1960). Cronbach’s alpha values were recorded for reliability of the perceived knowledge and attitude scales and subscales.

Statistical analysis was performed using the SPSS software, version 23. The normality of distribution of the perceived knowledge and attitude scores was confirmed via a calculation of the skewness and kurtosis; values for asymmetry and kurtosis between − 2 and + 2 are considered acceptable in order to prove normal univariate distribution (George 2011). These conditions consolidate the assumptions of normality in samples larger than 300 (Mishra et al. 2019). Accordingly, the Student *t*-test was used to check for an association between perceived knowledge and attitude scores and dichotomous variables (i.e., gender). Pearson correlation test was used to correlate two continuous variables. Significance was set at *p* < 0.05.

## Results

### Sociodemographic and other characteristics

The characteristics of the participants are displayed in Table 1. The mean age was 20.96 ± 2.67 years, with 58.8% females. The mean perceived knowledge score was 71.49 ± 8.44 (median = 73) and that of the attitude score 49.42 ± 3.87 (median = 50).

**Table 1:** Sociodemographic and cannabis use characteristics of 415 Lebanese university students

Variable	*N* (%)
**Gender**	
Male	171 (41.2%)
Female	244 (58.8%)
**Employment status**	
No	298 (71.8%)
Yes (part-time or full-time)	117 (28.2%)
**Previously tried cannabis**	
**for recreational purposes (yes)**	133 (32.0%)
**for medicinal purposes (yes)**	31 (7.5%)
**For both recreational and medicinal purposes (yes)**	28 (6.7%)
	**Mean (SD)**
**Age (in years)**	20.96 (2.67)
**Perceived knowledge about cannabis use scale score**	58.11 (14.57) (range 29–88)
**Attitudes about cannabis use scale score**	45.52 (6.35) (range 21–63)

Perceived knowledge is the participant accumulated information on cannabis use and its effects. Attitude is the individual’s thoughts and opinion on cannabis recreational and medical use. Both metrics were obtained from the respective scales created

Perceived knowledge about cannabis use scale score [min: 29, max: 145]

Attitudes about cannabis use scale score [min: 16, max: 80]

### Exploratory factor analysis (EFA) of the perceived knowledge about cannabis use scale items

The factor analysis was run over the total sample. All items concerning perceived knowledge about cannabis use could be extracted from the list except for item “How helpful do you think medical cannabis is for Memory problems” that showed a low communality (< 0.3). Items converged over a solution of four factors that had an Eigenvalue over 1, explaining a total of 64% of the variance (factor 1 = perceived knowledge about cannabis quality of life improvement; factor 2 = perceived knowledge about cannabis psychiatric use; factor 3 = perceived knowledge about cannabis risks; factor 4 = perceived knowledge about cannabis neurological use). The KMO value was 0.915, with a significant Bartlett’s test of sphericity (*p* < 0.001). A good Cronbach’s alpha value was found for the total scale (*α* = 0.78) (Table 2).

**Table 2:** Factor analysis of the perceived knowledge of cannabis use items using a promax rotation: loading of each item on its corresponding factor

	**Factor 1: Perceived knowledge about cannabis quality of life improvement**	**Factor 2: Perceived knowledge about cannabis psychiatric use**	**Factor 3: Perceived knowledge about cannabis risks**	**Factor 4: Perceived knowledge about cannabis neurological use**	**h2 communality**
1. How helpful do you think medical cannabis is for chronic pain control				0.843	0.728
2. How helpful do you think medical cannabis is for seizures control				0.790	0.627
3. How helpful do you think medical cannabis is for muscle spasms				0.696	0.621
4. How helpful do you think medical cannabis is for tremors found in various neurological conditions				0.845	0.703
5. According to you, how much can medical cannabis improve the quality of life in the following conditions: nausea and/or vomiting	0.692				0.572
6. According to you, how much can medical cannabis improve the quality of life in the following conditions: loss of appetite	0.873				0.704
7. According to you, how much can medical cannabis improve the quality of life in the following conditions: weight loss	0.825				0.614
8. According to you, how much can medical cannabis improve the quality of life in the following conditions: terminal illness	0.675				0.622
9. According to you, how helpful is medical cannabis in the following psychological and psychiatric conditions: post-traumatic stress disorder (PTSD)		0.721			0.708
10. According to you, how helpful is medical cannabis in the following psychological and psychiatric conditions: autism		0.486			0.553
11. According to you, how helpful is medical cannabis in the following psychological and psychiatric conditions depression		0.931			0.782
12. According to you, how helpful is medical cannabis in the following psychological and psychiatric conditions: general anxiety disorders		0.922			0.823
13. According to you, how helpful is medical cannabis in the following psychological and psychiatric conditions: difficulty sleeping (falling asleep, staying asleep)		0.662			0.593
14. To what extent do you think cannabis increases the risk for depression			0.676		0.529
15. To what extent do you think cannabis increases the risk for memory problems			0.631		0.409
16. To what extent do you think cannabis increases the risk for respiratory problems such as difficulty breathing			0.743		0.570
17. To what extent do you think cannabis increases the risk for car accidents if the driver is under the influence of cannabis			0.719		0.545
18. To what extent do you think cannabis increases the risk for drug overdose			0.865		0.768
19. To what extent do you think cannabis increases the risk for stroke			0.848		0.746
20. To what extent do you think cannabis increases the risk for diabetes			0.712		0.456
21. To what extent do you think cannabis increases the risk for heart attack			0.805		0.733
22. To what extent do you think cannabis increases the risk for addiction to cannabis itself			0.783		0.652
23. To what extent do you think cannabis increases the risk for addiction to drugs other than cannabis			0.859		0.752
24. To what extent do you think cannabis increases the risk for lung Cancer			0.791		0.605
25. To what extent do you think cannabis increases the risk for birth defects			0.804		0.600
26. To what extent do you think cannabis increases the risk for using drugs other than cannabis			0.854		0.758
27. To what extent do you think cannabis increases the risk for smoking Tobacco			0.721		0.511
28. To what extent do you think cannabis increases the risk for initiating drinking alcohol			0.777		0.642
Percentage of variance explained	10.0	11.8	32.4	9.8	
Cronbach’s alpha	0.884	0.927	0.965	0.884	

### Exploratory factor analysis (EFA) of the attitude about cannabis use scale’s items

The factor analysis was run over the total sample. All items concerning attitude about cannabis use could be extracted from the list except for items (people under 18 years old should not be using cannabis) and (driving a car while under the influence of cannabis should be a criminal offense) that showed a low communality (< 0.3). Items converged over a solution of two factors, explaining a total of 58.2% of the variance (factor 1 = negative attitudes about cannabis use; factor 2 = positive attitude for cannabis use). The KMO value was 0.915, with a significant Bartlett’s test of sphericity (*p* < 0.001). A good Cronbach’s alpha value was found for the total scale (*α* = 0.73) (Table 3).

**Table 3:** Factor analysis of the attitude about cannabis use items using a promax rotation

	**Factor 1 = Negative attitudes about cannabis use**	**Factor 2 = Positive attitude about cannabis use**	**h2 communality**
1. People have a good time when they use cannabis		0.507	0.499
2. Cannabis is a dangerous drug when used for non-medical conditions^a^	0.841		0.751
3. Cannabis use is a problem in our community^a^	0.804		0.653
4. You would be concerned if a friend or family is using cannabis^a^	0.671		0.481
5. You would use cannabis if a friend offered it to you		0.757	0.738
6. You would use cannabis if someone you do not know offered to you at a party		0.945	0.613
7. Using cannabis once a month is not dangerous	0.497		0.706
8. Most people who use cannabis will go on to use more dangerous drugs^a^	0.864		0.717
9. The benefits of using cannabis outweigh the harms and risks associated with its use		0.636	0.383
10. Using cannabis can lead people to become socially isolated^a^	0.741		0.506
11. It should be legal for people over the age of 18 to use cannabis		0.547	0.642
12. Many people who might use cannabis might be deterred by the possibility of getting a criminal conviction^a^	0.698		0.373
13. The sale of small amount of cannabis from one adult person to another should be considered a criminal offense^a^	0.797		0.634
14. It should not be illegal for a person to give another a small amount of cannabis		0.615	0.448
Percentage of variance explained	35.9	22.3	
Cronbach’s alpha	0.933	0.888	

^a^Items have reversed scoring. Items (people under 18 years old should not be using cannabis) and (driving a car while under the influence of cannabis should be a criminal offense) were removed because of low communality (< 0.3)

### Bivariate analysis

The bivariate analysis of categorical and continuous variables associated with the perceived knowledge and attitude about cannabis use scores are summarized in Tables 4 and 5. The results showed that greater perceived knowledge was found in male participants and in those who have tried cannabis for recreational purposes. Greater perceived knowledge was also associated with stronger attitude about cannabis use and older age. Furthermore, a greater mean attitude about cannabis use was significantly found in male participants and in those who have tried cannabis for recreational or medicinal purposes.

**Table 4:** Bivariate analysis of categorical factors associated with the perceived knowledge and attitude scores

Variable	Perceived knowledge about cannabis (SD)	Attitude about cannabis use (SD)
**Gender**		
Male (*N* = 171)	69.94 (8.00)	42.88 (5.06)
Female (*N* = 244)	67.82 (8.73)	41.60 (4.46)
*P*	**0.012**	**0.007**
Effect size	0.253	0.268
**Employment status**		
No (*N* = 298)	68.27 (8.53)	42.22 (4.64)
Yes (*N* = 117)	69.77 (8.32)	41.89 (5.04)
*P*	0.106	0.526
Effect size	0.178	0.068
**Tried cannabis for recreational purposes**		
No (*N* = 282)	67.69 (8.98)	41.63 (4.71)
Yes (*N* = 133)	70.81 (6.90)	43.17 (4.68)
*P*	** < 0.001**	**0.002**
Effect size	0.389	0.328
**Tried cannabis for medicinal purposes**		
No (*N* = 384)	68.57 (8.71)	41.99 (4.71)
Yes (*N* = 31)	70.34 (4.74)	43.84 (4.97)
*P*	0.07	**0.037**
Effect size	0.253	0.382

Numbers in bold indicate significant *p*-values; Student *t* test was used to compare 2 means. Effect size refers to Cohen’s *d* value

**Table 5:** Bivariate analysis of continuous factors associated with the perceived knowledge and attitude against cannabis use scores

Variable	Perceived knowledge about cannabis	Attitude about cannabis use
Perceived knowledge	1	
Attitude against cannabis use	*r* = 0.189; ***p* < 0.001**	1
Age	*r* = 0.105; ***p* = 0.033**	*r* = 0.014; *p* = 0.781

Numbers in bold indicate significant *p*-values; Pearson’s correlation test was used to correlate two continuous variables

## Discussion

### Scales’ factorial validity

The factor analysis of the perceived knowledge about cannabis use items resulted in 4 factors (Table 2), and the analysis of the attitude about cannabis use items resulted in 2 factors (Table 3). These two scales, on another hand, showed good Cronbach’s alpha values. The internal consistency is considered acceptable above the commonly suggested threshold of 0.70 (Furr 2021; Hair et al. 2011). These scales may be completed in 5–7 min; the questions are simple to comprehend, making them useful tools for future research.

### Correlates of perceived knowledge about and attitude about cannabis use

In this study, greater perceived knowledge and stronger attitudes about cannabis were found in male participants compared to women. A previous study showed that male reported greater cannabis use over time (Hemsing and Greaves 2020). To the best of our knowledge, no previous research has looked at the relationship between gender and cannabis perceived knowledge about and attitudes about cannabis. Many possibilities can explain this finding. This can be explained by the fact that men use cannabis more frequently than women (Carliner et al. 2017), and they have greater perceived knowledge about this substance. Furthermore, when males use the substance more frequently, they are more likely to be subject to withdrawal symptoms, commonly seen among users of the substance (Bahji et al. 2020). As a result, their attitude towards cannabis use might become more negative. In addition, men are more likely to initiate cannabis use at younger age than women (Pope et al. 2003). Another possible explanation for the existing sex differences in cannabis use may reflect women’s increased perceptions of risks associated with regular use (Cuttler et al. 2016). However, future research on biological and psychosocial mechanisms underlying cannabis-related sex differences are needed to better understand this association.

Greater perceived knowledge was also associated with a stronger attitude about cannabis use. Our results are consistent with previous findings correlating greater knowledge to better attitude about cannabis in athletes (Zeiger et al. 2020a). We also found that having tried cannabis for recreational or medicinal purposes was significantly associated with greater perceived knowledge. This can be explained by the Knowledge-Attitude-Behavior model, which postulates the temporal ordering, as follows: “As knowledge accumulates in a health behavior domain, changes in attitude are initiated. Over some period of time, changes in attitude accumulate, resulting in behavioral change” (Baranowski et al. 2003). In terms of cannabis use, the triad of knowledge, attitudes, and behavior has not been widely explored, although past studies examining cannabis use in adolescents have followed a harms-avoidance approach (Asbridge et al. 2014). Adolescent drug knowledge, pro-drug attitudes, and adolescent drug use were all assessed in research on parenting methods. Both drug knowledge and drug use were predicted by pro-drug attitudes (Zeiger et al. 2020b). Another study among adolescents found that drug use education influenced substance use attitudes, resulting in lower cigarette and cannabis use. However, the growing acceptance of cannabis use among the general public in the USA is leading to increased use, suggesting a review of the knowledge-attitudes-behavior paradigm (Zeiger et al. 2020b). It is noteworthy to draw the attention to one important issue: we cannot separate/differentiate attitude against cannabis use from attitude towards cannabis use since some people might be against cannabis use for recreational purposes, but they lean towards cannabis use for medicinal purposes. Future studies are needed to differentiate both types of attitudes.

On another hand, we found that a greater mean attitude about cannabis use in those who have tried cannabis for medicinal purpose. This result aligns with the fact that medical cannabis has gotten a lot of attention due to its possible beneficial effects on chronic pain, nausea, fibromyalgia, inflammatory bowel disease, and other difficult-to-treat conditions (Zeiger et al. 2020c). Participants in a previous study overestimated the medicinal efficacy of cannabis, while underestimating the risks (Kruger et al. 2020). Although there is a lack of evidence on its therapeutic benefits, there is also lack of perceived knowledge about its negative health consequences. There is substantial evidence that cannabis has negative effects in many contexts such as on the respiratory (Tetrault et al. 2007; Moore et al. 2005; Pletcher et al. 2012) (especially inhaled cannabis (Loflin and Earleywine MJCjortCRcdltrR, 2015)), as well as the reproductive (Kolodny et al. 1974; Maccarrone et al. 2021; Hsiao 2018), gastrointestinal (Sullivan 2010; Naftali et al. 2014; Gotfried et al. 2020), and immunologic (Svrakic et al. 2012; Friedman et al. 2003) systems.

### Limitations

The data’s cross-sectional nature limits the ability to make causal conclusions and the use of a self-administered questionnaire containing some unfamiliar terms poses a risk for information bias. Besides, an under/over-estimation and the misunderstanding of a question could be experienced by a participant, leading to an information bias. There is also a risk for selection bias, given the sample was recruited from a single university, with a female predominance. A residual confounding bias might be present since not all factors known to influence perceived knowledge and attitude about cannabis use were considered in this paper. Further studies are needed in order to validate it among the general population and assess more psychometric properties of these scales.

## Conclusion

This study was able to create and validate two scales to assess perceived knowledge about and attitude about cannabis use in Lebanese university students. The primary results showed acceptable psychometric properties. We hope these scales will be useful in future epidemiological studies.

## Data Availability

All data generated or analyzed during this study are not publicly available to maintain the privacy of the individuals’ identities. The dataset supporting the conclusions is available upon request to the corresponding author.

## Abbreviations

THC: Delta-9-tetrahydrocannabinol
HIV: Human immunodeficiency virus
